# Celastrol suppresses bone destruction in rheumatoid arthritis by inhibiting ALOX5 expression in macrophages via the NF-κB pathway

**DOI:** 10.1038/s41598-025-33001-x

**Published:** 2025-12-17

**Authors:** YiQing Chen, Zihan Wang, YanYu Chen, XiaoJing Liu, LongXiao Liu, ZhiKun Tu, Qingwen Tao, Yuan Xu

**Affiliations:** 1 National Center for Integrative Medicine, Department of TCM Rheumatism, China-Japan Friendship Hospital, No. 2 Yinghua East Street, Chaoyang District, Beijing, People’s Republic of China; 2 Beijing University of Chinese Medicine, Beijing, People’s Republic of China

**Keywords:** Celastrol, Rheumatoid arthritis, Bone destruction, ALOX5, NFκB, Computational biology and bioinformatics, Diseases, Genetics, Immunology, Rheumatology

## Abstract

Rheumatoid arthritis (RA) is a complex and highly disabling chronic autoimmune disease. As the disease progresses, patients often develop complications such as joint destruction and cardiovascular diseases, posing significant threats to human health. Celastrol, a major bioactive compound extracted from the traditional Chinese herb Tripterygium wilfordii Hook. f., exhibits potent immunomodulatory and anti-inflammatory properties. However, the specific mechanisms underlying its protective effects against bone destruction in RA remain poorly understood. To elucidate its potential therapeutic mechanisms, this study retrieved three gene expression datasets—GSE55235, GSE93777, and GSE200815—from the Gene Expression Omnibus (GEO) database. The primary molecular targets of celastrol were obtained from the HERB and TCMSP platforms. Functional mechanisms associated with these targets were explored using gene set variation analysis (GSVA) and weighted gene co-expression network analysis (WGCNA). Furthermore, molecular docking, immune infiltration analysis, and single-cell RNA sequencing analysis were employed to investigate the role of key target genes. In this study, thirteen potential target genes of celastrol in RA have been identified, including *ADAMTS5*, *AGTR1*, *ALOX5*, *CTSB*, *MMP3*, *MMP9*, *MYC*, *TNF*, *ITGA4*, *ITGB7*, *MMP1*, *MMP13*, and *PPARG*. Among these, *ALOX5* was found to significantly promote MMP3 protein expression, based on which a regulatory model with high predictive power was constructed. GSVA analysis revealed that the TNF-NFκB pathway was significantly activated in RA and exhibited a strong positive correlation with *ALOX5* expression. Further experimental analysis demonstrated that knockdown of *ALOX5* and its shared transcription factor with *MMP2* resulted in a significant downregulation of both genes and inhibition of TNF-NFκB pathway activity. Single-cell transcriptomic analysis showed that *ALOX5* was predominantly expressed in macrophages, and the AddModuleScore of celastrol-targeted genes in this cell type was significantly higher than in other cell types, suggesting that macrophages may serve as key effector cells in celastrol-mediated treatment of RA. Celastrol might attenuate RA bone destruction by inhibiting the expression of the *ALOX5* gene in macrophages, thereby suppressing the activation of the NF-κB pathway and subsequently reducing the production of matrix metalloproteinases.

**Supplementary Information:**

The online version contains supplementary material available at 10.1038/s41598-025-33001-x.

## Introduction

Rheumatoid arthritis (RA) is a chronic autoimmune disease characterized by persistent joint inflammation and progressive bone destruction^1,2^. The global prevalence of RA ranges from approximately 0.24% to 1%, with a significantly higher incidence observed in women than in men^3^. The pathogenesis of RA is complex and involves the overproduction of multiple pro-inflammatory cytokines, including TNF-α, IL-6, and IL-17, which induce RANKL expression and subsequently promote osteoclast activation and bone resorption^4–7^. In addition, macrophages contribute to joint destruction by releasing inflammatory mediators and matrix metalloproteinases (MMPs), further amplifying the inflammatory cycle^8–11^. Elevated expression of various MMPs, such as MMP-1, MMP-3, MMP-9, and MMP-13, has been consistently observed in the synovial tissues of RA patients. These enzymes degrade extracellular matrix components of articular cartilage and bone, leading to structural joint damage and functional impairment. Notably, studies have demonstrated that inhibition of MT1-MMP synergizes with anti-TNF therapy to delay disease progression in collagen-induced arthritis models in mice^12^. Despite advances in treatment strategies—including conventional synthetic DMARDs (csDMARDs), biologic DMARDs (bDMARDs), and targeted synthetic DMARDs (tsDMARDs)—which have shown efficacy in controlling inflammation, a significant proportion of patients exhibit inadequate responses or develop long-term safety concerns, particularly an increased risk of infections^13–16^. Therefore, the development of novel therapeutic strategies that are more effective, safer, and cost-effective remains a critical objective in current RA research.

Celastrol, an immunosuppressive compound derived from the traditional Chinese herb Tripterygium wilfordii, has been increasingly recognized for its ability to modulate NF-κB and Notch1 signaling pathways, thereby inhibiting the differentiation of macrophages into pro-inflammatory phenotypes and reducing the production of inflammatory cytokines and the progression of RA^17,18^. However, systematic investigations and conclusive evidence regarding the therapeutic effects of celastrol on specific clinical manifestations of RA, such as joint inflammation and bone destruction, remain limited.

This study aims to investigate the impact of celastrol on RA and RA-associated bone destruction by integrating high-throughput sequencing with single-cell transcriptomic analysis, with a focus on elucidating its underlying mechanisms of action.

## Methods

### Data acquisition and differential expression analysis

The gene chip datasets GSE55235 (normal = 10, RA = 10) and GSE93777 were retrieved from the GEO database (http://www.ncbi.nlm.nih.gov/geo), where GSE93777 includes 448 whole blood transcriptome and immune cell samples (Supplymentory_Table 1). Probe and gene names were aligned according to the GPL platform annotations. The count matrix of GSE55235 was log-normalized, and a linear model was constructed using the limma package in R. The model was fitted using the lmFit function and subsequently subjected to Bayesian statistical analysis using the eBayes function. Differentially expressed genes were identified by setting the log_2_FC threshold to 1 and the p-value threshold to 0.05. Visualization of gene expression patterns was performed using volcano plots and heat maps, generated with the ggplot2 and pheatmap packages, respectively.

### Enrichment analysis

Differential gene analysis was performed using the clusterProfiler package in R to conduct GO and KEGG pathway enrichment analyses^19–21^. The GO categories, including biological processes (BP), molecular functions (MF), and cellular components (CC), were adjusted for multiple testing using the Benjamini-Hochberg method, with a threshold of FDR < 0.05. The top 5 significantly enriched results were selected for visualization in the GO enrichment bubble plots, and the top 15 results with the most significant adjusted p-values were illustrated in the KEGG enrichment bar plots^19,22^. Both types of plots were generated using the ggplot2 package.

### Weighted gene co-expression network analysis (WGCNA)

A weighted gene co-expression network was constructed using the WGCNA package on the GSE55235 dataset to identify gene modules significantly associated with phenotypes. Initially, the optimal soft threshold power was determined using a scale-free topological model. Subsequently, a weighted adjacency matrix was generated and transformed into a topological overlap matrix (TOM) to evaluate the overall correlation among genes. Hierarchical clustering, based on TOM dissimilarity, was performed to group genes with similar expression profiles into distinct modules, with module eigengenes representing each module’s overall expression patterns. The correlation between modules and phenotypes was calculated to identify modules significantly associated with the phenotypes, and gene significance (GS) and module membership (MM) were assessed to identify pivotal driver genes. Finally, the network structure was visualized using a TOM heat map and a module cluster tree.

### Identification of key genes targeted by Celastrol

The potential target genes of Celastrol were identified using the HERB database (http://herb.ac.cn/). The SMILES chemical structure of tripterine was retrieved from the PubChem database (https://pubchem.ncbi.nlm.nih.gov/) and input into the Swiss Target Prediction database (http://www.swisstargetprediction.ch/) to predict potential target information. Subsequently, the target genes obtained from both sources were consolidated, and gene names were standardized to official gene symbols using the UniProt database (https://www.uniprot.org/). Target genes associated with RA were extracted from the DisGeNET database (https://www.disgenet.org/). An intersection analysis was then conducted among the predicted targets of tripterine, RA-related targets, RA differentially expressed genes identified in prior screenings, and key module genes derived from WGCNA to identify potential key target genes of tripterine for treating RA.

### Correlation analysis of key genes with clinical phenotypes

Box plots depicting the expression levels of key target genes across various samples from GSE55235 were generated using the ggplot2 package. To further investigate the relationships between the expression of key genes, clinical indicators, and MMP3 protein expression levels, the external dataset GSE93777 was used for validation. Pearson correlation coefficient analysis was performed to examine the associations between key genes and clinical indicators, with the results visualized using heatmaps. Subsequently, multiple linear regression models were developed based on the correlation analysis findings to assess the impact of different factors on MMP3 protein expression levels. The MMP3 protein levels were categorized as high or low based on their median values, and linear models were constructed using the lm function. The model summaries were then used to extract regression coefficients, standard errors, and significance levels. Forest plots were created to illustrate the estimates of variables along with their corresponding 95% confidence intervals. Hierarchical partitioning was conducted to determine the relative contributions of the variables to the variance explained within the model. Independent R^2^ values and percentage contributions (I.perc) were calculated for each factor, and stacked histograms were used to visualize the explanatory power of various variable categories. Additionally, correlation plots between core genes and the gene expression of different MMPs families were generated using the Spearman rank correlation coefficient. Statistical significance (*p* < 0.05) was indicated in the results plotted using ggplot2.

### Construction of ROC curves

Receiver Operating Characteristic (ROC) curves were constructed based on the core genes identified in prior screening to systematically evaluate the classification performance of each predictive model.

### Genome set variation analysis (GSVA)

To investigate the functional implications of these core genes at the pathway level, GSVA was performed on samples from the GSE93777 dataset using the Hallmark (H) dataset from the MSigDB database (https://www.gsea-msigdb.org/gsea/msigdb/index.jsp) as a reference background. Subsequently, differences in GSVA scores between sample groups with high and low gene expression levels were evaluated using the limma package. Pathways with GSVA scores that increased significantly (t > 2) were considered activated in RA, whereas those with scores that decreased significantly (t < − 2) were considered inhibited. To elucidate potential relationships between core genes and key pathways, Spearman’s rank correlation analysis was performed, and scatter plots were generated to display statistically significant results (*p* < 0.05) with correlation coefficients ≥ 0.4. Furthermore, scatter plots illustrating correlations between GSVA scores for pivotal pathways and clinical markers of RA (Erythrocyte Sedimentation Rate - Disease Activity Score in 28 joints(ESRDAS28), C-reactive Protein - Disease Activity Score in 28 joints(CRPDAS28)) were generated to explore their biological relevance in disease progression.

### Analysis of upstream transcription factors of core genes

To investigate the upstream regulatory mechanisms of core genes, we utilized the TRRUST database (https://www.grnpedia.org/trrust/) to identify potential upstream transcription factors. This database compiles a substantial number of experimentally validated regulatory relationships between human and mouse transcription factors and their target genes, ensuring high reliability. By leveraging the regulatory data from the database, we identified transcription factors strongly associated with core genes and subsequently constructed transcription factor-target gene regulatory networks.

### Analysis of gene knockout data using GPSAdb

To identify potential upstream transcription factors of core genes and explore their regulatory effects, we conducted a systematic search using the GPSAdb database (https://www.gpsadb.com/). This database integrates 3048 RNA-seq datasets covering 1458 genes subjected to genetic perturbation via siRNA, shRNA, CRISPR/Cas9, or CRISPRi technologies. A total of 6096 new perturbed gene sets were constructed from these datasets^23^.We retrieved the transcription factor information associated with the core genes and generated the corresponding differential expression matrices following gene knockdown. Differential expression analysis was performed with the criteria of |log₂FC| ≥ 0.5 and *p* < 0.05, and the results were visualized via volcano plots to pinpoint biologically significant regulatory factors. Subsequently, GSEA was conducted to functionally annotate the target genes of the identified transcription factors, thereby elucidating their potential involvement in pertinent pathways and biological processes.

### Molecular Docking analysis

The PDB files in Celastrol 3D format were retrieved from PubChem (https://pubchem.ncbi.nlm.nih.gov/) and subsequently converted to the MOL2 format using OpenBabel 2.4.1. The PDB files of the core genes were obtained from the UniProt website (https://www.uniprot.org/) based on the criteria of higher resolution (lower resolution values) and longer sequence length. Ten semi-flexible molecular docking simulations were conducted using AutoDock 4.2.x, and the result set with the lowest binding energy was selected for visualization using PyMOL.

### Immune infiltration analysis

CIBERSORT is a computational method for characterizing the complex cellular composition of tissues based on gene expression profiles^24^. The CIBERSORT algorithm was applied to calculate the proportions of 28 immune cell types in whole blood tissues from rheumatoid arthritis (RA) and healthy populations within the GSE93777 dataset^25^, and the Spearman algorithm was employed to assess the correlations between key genes and immune cells, as well as among immune cells.

### Consensus clustering

To identify the molecular subtypes of the samples in the GSE93777 dataset, we performed consensus clustering analysis using the K-means algorithm on the gene expression data from 232 whole-tissue samples. The top 5000 genes with the highest median absolute deviation (MAD) were selected and normalized to median centrality; then the ConsensusClusterPlus package was employed to perform consensus clustering, with the maximum number of clusters set to 6, sampling repeated 500 times, and the Pearson correlation coefficient used as the distance metric. The optimal number of clusters was determined based on the consistency matrix and cluster consensus score. Finally, the consistency matrix and cluster labels were output, and the hierarchical clustering tree was generated to visualize the sample distribution. Further analysis was conducted to examine the expression differences of core genes and MMPs family genes across different subgroups.

### Single-cell analysis

Single-cell expression data from 4 RA patients in the GSE200815 dataset were selected, and the parameters were set to min.cells = 5 and min.features = 300 to read the expression profiles, yielding an initial expression matrix of 20,977 × 48,297. The object was screened to exclude low-expression cells using the criteria 200 ≤ nFeature_RNA ≤ 10,000, 500 ≤ nCount_RNA ≤ 100,000, percent_mito < 10%, percent_hb < 1%, and expression in at least 3% of the cells, resulting in a final single-cell object of 20,977 × 15,843. The data were normalized using the LogNormalize method with a scale factor of 10,000, followed by sequential PCA analysis via ScaleData and RunPCA. Batch correction of the PCA results was performed using Harmony, and dimensionality reduction was conducted on the first 15 principal components using the UMAP algorithm. Cell clustering analysis and UMAP downscaling were sequentially performed using FindNeighbors, FindClusters, run_harmony, and RunUMAP, and cell annotation was conducted based on references. Stacked histograms of cell expression were generated using ggplot2.

### Differential gene expression analysis

The parameters were set to min.pct = 0.25 and logfc.threshold = 0.25, and differentially expressed genes in each cell were identified using the FindAllMarker function.

### AddModuleScore

The Celastrol-interacting genes identified in Section ‘Identification of Key Genes Targeted by Celastrol’ were used as a customized gene set, and the average expression value of each gene in each cell was calculated to assess the responsiveness of different cells to celastrol.

### Gene set enrichment analysis (GSEA)

GSEA was employed to evaluate the enrichment of gene sets in biological processes and the expression levels of core genes in the corresponding pathways.

## Results

### Enrichment analysis and WGCNA

**Fig. 1:**
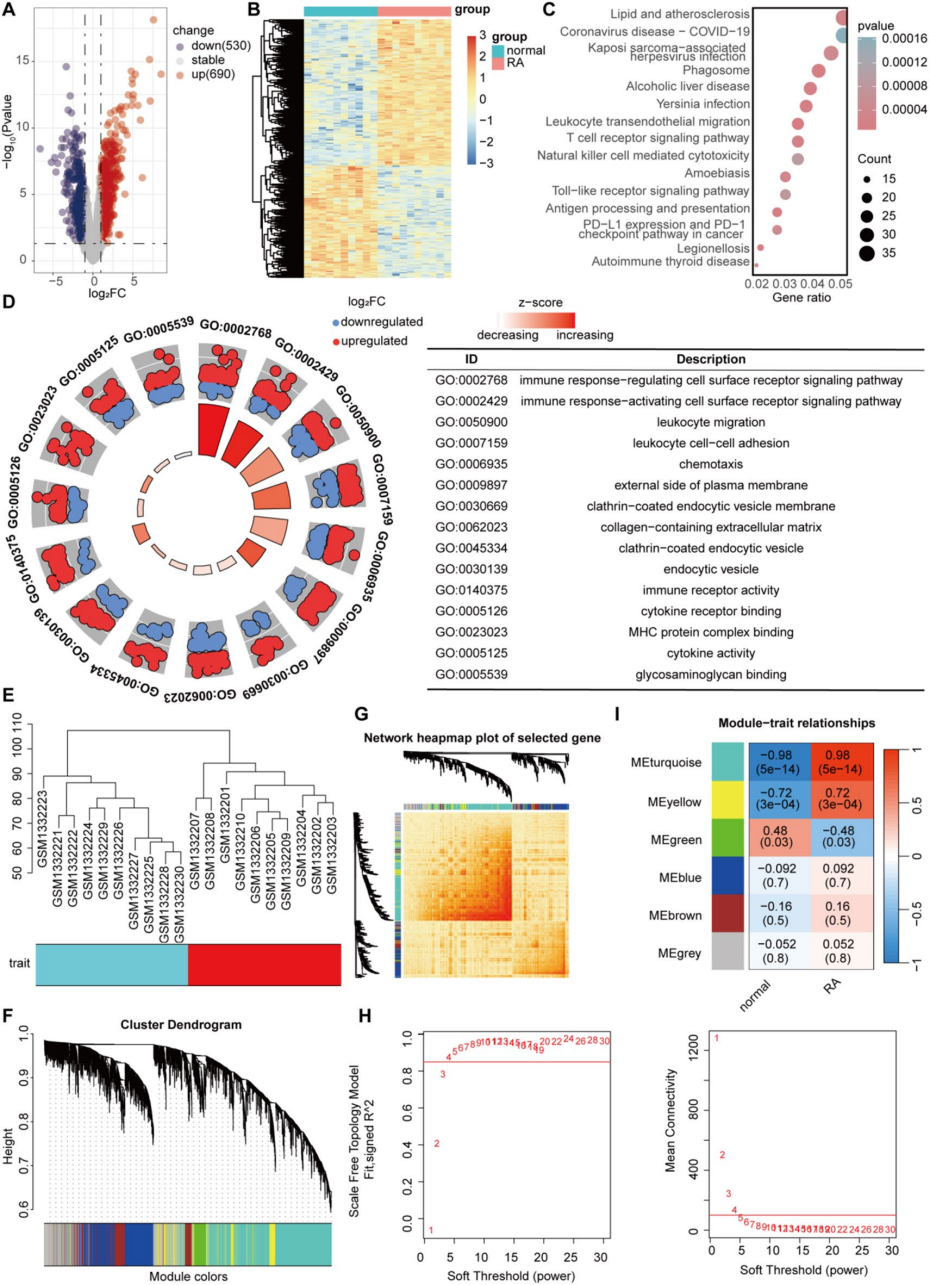
Data acquisition and enrichment, WGCNA ^19,22^. (**A**) Volcano plot for differential expression analysis. (**B**) Heat map for differential expression analysis. (**C**) KEGG enrichment. (**D**) GO enrichment. (**E**) Sample and module dendrogram of GSE55235. (**F**) Cluster dendrogram. (**G**) Topological overlap matrix of DEGs. (**H**) Analysis of scale-free index and mean connectivity of various soft thresholds. (**I**) WGCNA analysis for RA. DEGs, Differentially Expressed Genes; GO, Gene Ontology Enrichment Analysis; KEGG, Kyoto Encyclopedia of Genes and Genomes; RA, rheumatoid arthritis; WGCNA, weighted gene co-expression network analysis.

We selected 10 normal control samples and 10 RA samples from peripheral blood mononuclear cells (PBMCs) within the GSE55235 dataset, which included information on 12,548 genes. Differential expression analysis was performed with the criteria of |log_2_FC| ≥ 1 and *p* < 0.05, yielding 1220 differentially expressed genes (DEGs), including 690 upregulated and 530 downregulated genes (Fig. 1A,B). KEGG analysis revealed enrichment in pathways such as the T-cell receptor signaling pathway and natural killer cell mediated cytotoxicity (Fig. 1C)^19,22^. Circular plots displayed GO enrichment results, indicating that the RA group was primarily associated with immune response and inflammatory factors (Fig. 1D).

**Fig. 2:**
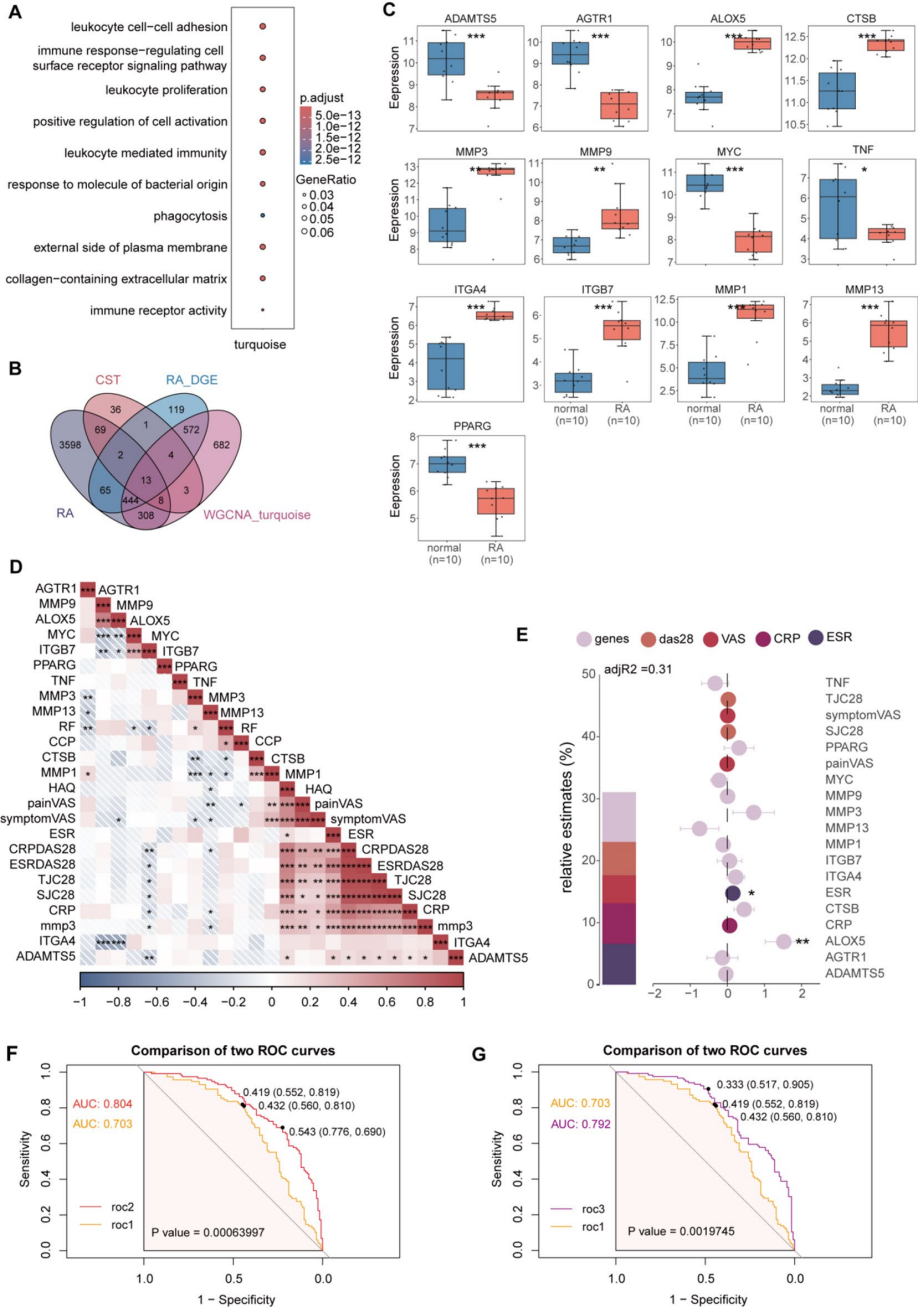
Identification of key genes. (**A**) GO enrichment of genes in MEturquoise. (**B**) The Overlapping genes between DEGs and the MEturquoise module. (**C**) Boxplot of key genes. (**D**) Corplot. (**E**) Multiple linear regression models. (**F**) Comparison of roc1 and roc2. (**G**) Comparison of roc1 and roc3. CRP, C-reactive protein; CRPDAS28, C-reactive Protein - Disease Activity Score in 28 joints; DEGs, Differentially Expressed Genes; ESR, Erythrocyte Sedimentation Rate; ESRDAS28, Erythrocyte Sedimentation Rate - Disease Activity Score in 28 joints; GO, Gene Ontology Enrichment Analysis; HAQ, Health Assessment Questionnaire; ROC, receiver operating characteristic; SJC28, swollen joint count for 28 joints; TJC28, tender joint counts for 28 joints; VAS, Visual Analog Scale.

The main biological processes involved in the RA group included immune response − regulating cell surface receptor signaling pathway, immune response − activating cell surface receptor signaling pathway, leukocyte migration, leukocyte cell − cell adhesion, chemotaxis; cellular components included external side of plasma membrane, clathrin − coated endocytic vesicle membrane, collagen − containing extracellular matrix, clathrin − coated endocytic vesicle, endocytic vesicle; molecular functions included immune receptor activity, cytokine receptor binding, MHC protein complex binding, cytokine activity, glycosaminoglycan binding. WGCNA was employed to determine the soft threshold of 6 (Fig. 1E–H). Under this threshold, the network constructed showed a significant correlation between the cyan module and RA (Fig. 1I, module-trait correlation analysis revealed a correlation coefficient of 0.98 and a P value < 0.001 for the cyan module with RA). These results suggest that genes in the cyan module may play a significant role in the pathogenesis of RA. KEGG analysis of the cyan module revealed significant enrichment of genes in processes such as immune response and cell receptors (Fig. 2A).

### Identification of key genes involved in the effects of Celastrol

**Fig. 3:**
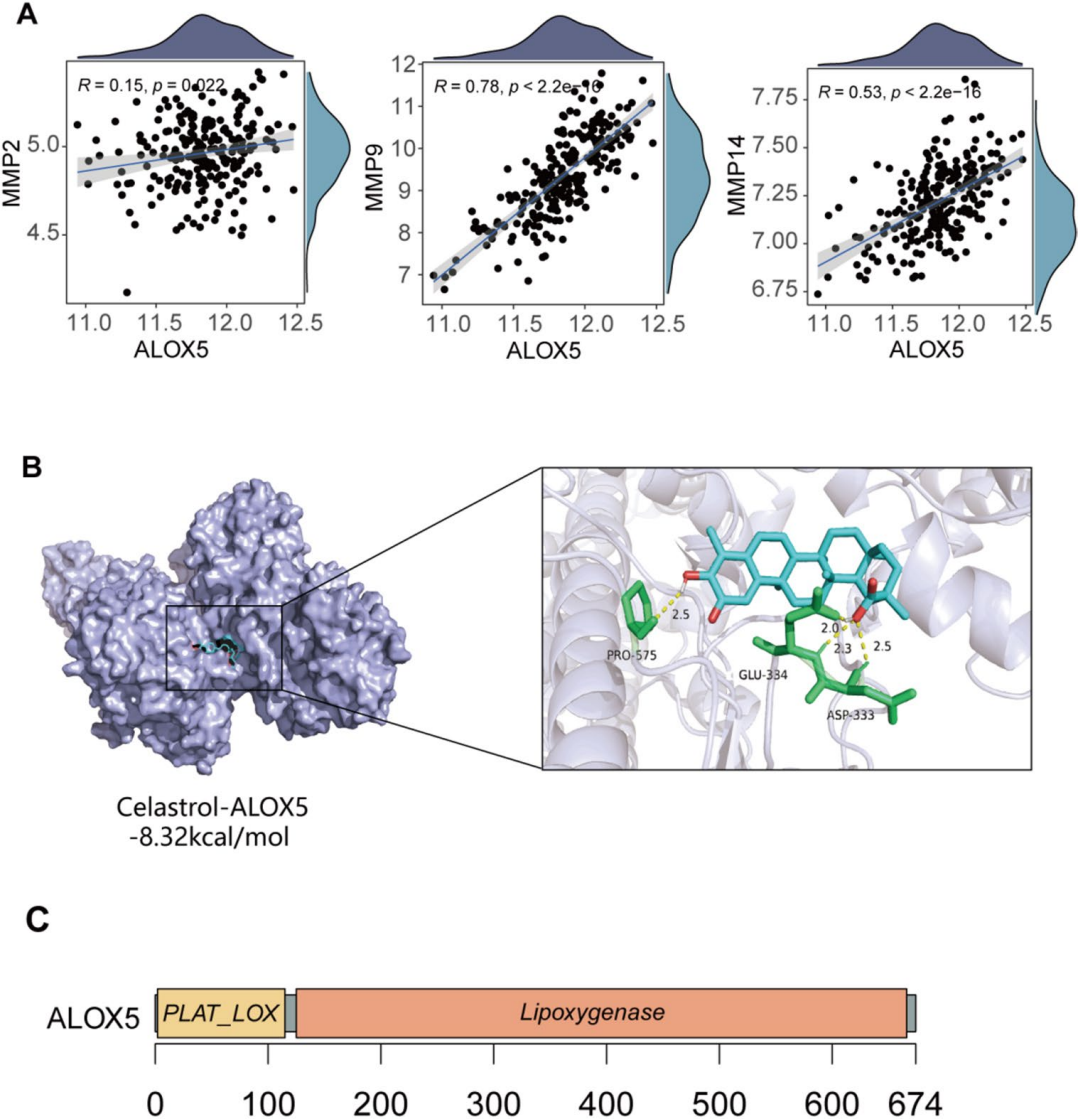
Molecular docking of CST and ALOX5. (**A**) Corplot of *ALOX5* and MMPs. (**B**) Molecular docking of CST and *ALOX5*. (**C**) Structural characterization of the *ALOX5* protein. CST, Celastrol; MMPs, matrix metalloproteinases

The intersection of Celastrol-target genes, turquoise module genes, RA differentially expressed genes, and RA-associated genes were taken, and 13 intersecting genes(*ADAMTS5*, *AGTR1*, *ALOX5*, *CTSB*, *MMP3*, *MMP9*, *MYC*, *TNF*, *ITGA4*, *ITGB7*, *MMP1*, *MMP13*, *PPARG*) were selected as key genes (Fig. 2B). Box plots displayed their expression profiles in GSE55235 (Fig. 2C). Subsequently, the correlation between these 13 genes and clinical manifestations was explored using the external dataset GSE93777 (Fig. 2D). Figure 2E showed the impact of these 13 genes and various clinical indicators on MMP3 protein expression levels, with MMP3 protein levels as the dependent variable. The results indicated that *ALOX5* and Erythrocyte Sedimentation Rate(ESR) significantly positively influenced MMP3 protein levels. Subsequently, different ROC models were constructed, and samples were divided into high and low groups based on the median expression level of MMP3 protein. The results showed that the AUC values of the predictive models constructed with *ALOX5*, *MMP3*, *MMP2*, *MMP9*, *MMP14*, tender joint counts for 28 joints(TJC28), swollen joint count for 28 joints(SJC28), C-reactive protein(CRP), ESR (Fig. 2F, AUC = 0.804) and *ALOX5*, TJC28, SJC28, CRP, ESR (Fig. 2G, AUC = 0.792) were significantly higher than those constructed solely with TJC28, SJC28, CRP, ESR (AUC = 0.703). Particularly, the model with *ALOX5*, *MMP3*, *MMP2*, *MMP9*, *MMP14*, TJC28, SJC28, CRP, ESR exhibited high diagnostic performance. Scatter plots displayed the mechanistic metalloproteinase-encoding genes significantly correlated with *ALOX5* expression (Fig. 3A). Collectively, these findings establish *ALOX5* as a central regulatory hub and a promising therapeutic target in RA.

### Molecular docking of CST and *ALOX5*

To predict the potential direct binding capacity between celastrol and *ALOX5*, molecular docking was performed. The results demonstrated that celastrol binds to *ALOX5* with a binding energy of -8.32 kcal/mol, indicating a favorable interaction (Fig. 3B). Triptolide forms a binding pocket through interactions with ASP at position 333, GLU at position 334, and PRO at position 575 of *ALOX5*. As depicted in Fig. 3C, *ALOX5* is an enzyme composed of 674 amino acids and primarily consists of two domains: the PLAT_LOX domain (2-118) and the Lipoxygenase domain (119–674).

### *ALOX5* operates primarily through the NFκB pathway

**Fig. 4:**
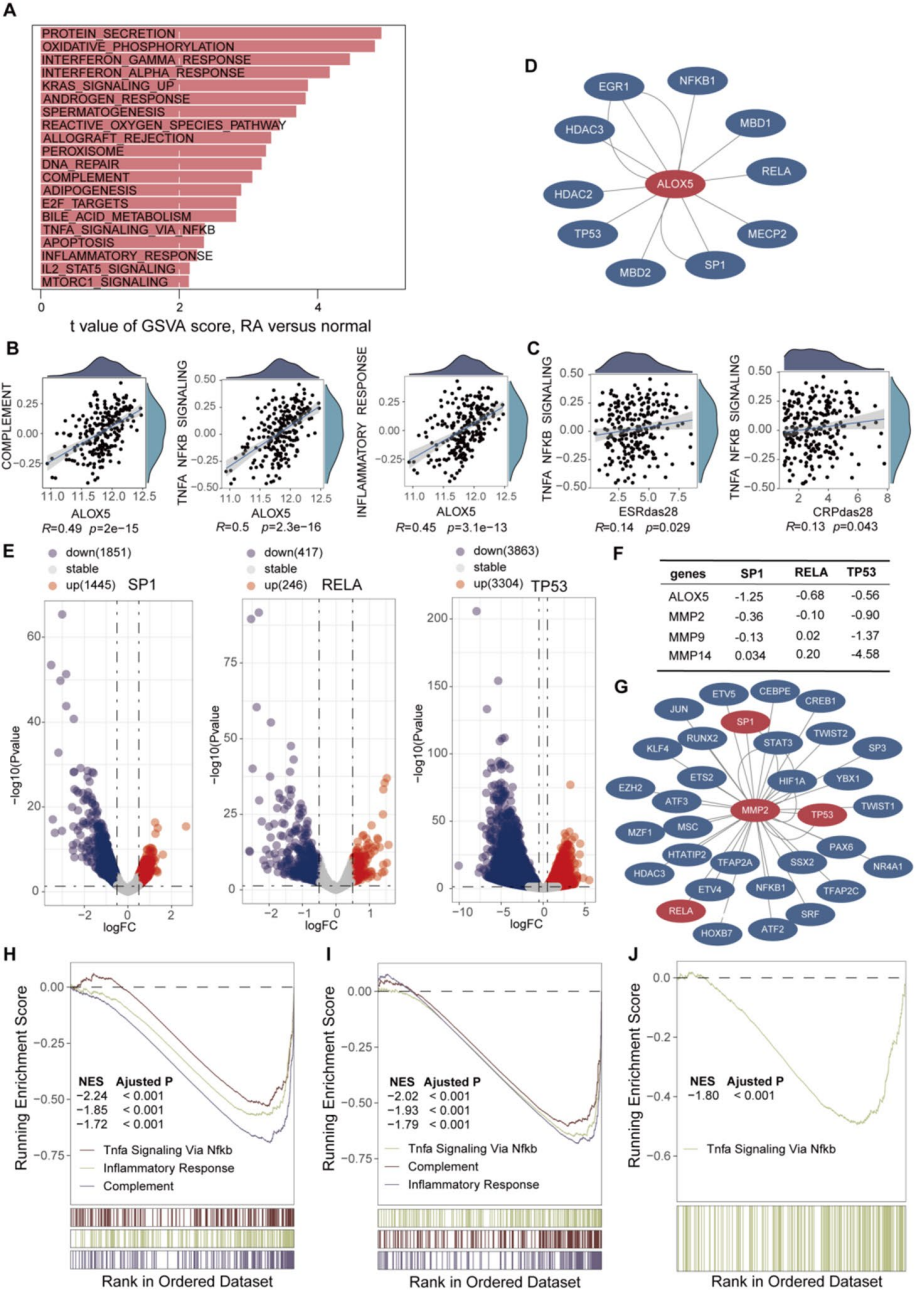
GSVA and GSEA. (**A**) GSVA analysis for GSE93777. (**B**) Corplot of *ALOX5* and complement, TNF-NFKB pathway, inflammatory response. (**D**) Transcriptional Regulatory Network of *ALOX5*. (**E**) Analysis of Gene Knockout Data Using GPSAdb. (**F**) Log_2_FC of *ALOX5*, MMPs in Gene Knockout Data. (**G**) Transcriptional Regulatory Network of *MMP2*. (**H**) GSEA analysis for *SP1* knockout data. (**I**) GSEA analysis for *RELA* knockout data. (**J**) GSEA analysis for *TP53* knockout data. GSEA, Gene Set Enrichment Analysis; GSVA, gene set variation analysis; NFκB, Nuclear Factor kappa-light-chain-enhancer of activated B cells.

GSVA enrichment analysis revealed significant activation of multiple biological pathways in the RA group, including protein secretion, oxidative phosphorylation, interferon-gamma response, and Kras signaling (up-regulated) pathways (Fig. 4A), suggesting their involvement in inflammatory and immune regulatory processes. Further analysis demonstrated that *ALOX5* expression was significantly correlated with the complement pathway, TNF-NFκB pathway, and inflammatory response pathway (Spearman’s *R* = 0.49, 0.50 and 0.45, respectively; all *P* < 0.001). Notably, the strongest correlation was observed between *ALOX5* and the TNF-NFκB pathway (Fig. 4B), indicating a potential central regulatory role of *ALOX5* in this pathway.

According to standard statistical criteria, Spearman’s R values ranging from 0.4 to 0.6 indicate moderate correlations. In this study, the correlation coefficient between *ALOX5* and the TNF-NFκB pathway was 0.50, suggesting a moderate positive association. Moreover, increased activity of the TNF-NFκB pathway was significantly associated with elevated ESRDAS28 and CRPDAS28 levels (*R* = 0.14 and 0.13, respectively; *P* < 0.001) (Fig. 4C). Although these correlations were relatively weak, they remained statistically significant in the large sample size, potentially reflecting an underlying molecular mechanism of disease activity in RA. Collectively, these findings support the hypothesis that *ALOX5* primarily contributes to the inflammatory process in RA through modulation of the TNF-NFκB signaling pathway.

### Upstream mechanisms governing ALOX5 expression

*ALOX5* has 10 upstream transcription factors (Fig. 4D), among which RELA and NFκB1 are key factors in the NF-κB pathway, and data from GPSAdb revealed that the absolute logFC value of *ALOX5* was less than 0.5 after knockout of *RELA*, *SP1*, and *TP53* genes (Fig. 4E). Following *ALOX5* knockout, the expression of *MMP2* was reduced (Fig. 4F), leading to the construction of a transcription factor regulatory network upstream of *MMP2*, which revealed that *RELA*, *SP1*, and *TP53* jointly regulate the expression of both genes (Fig. 4G). GSEA enrichment analysis showed that the TNF-NFκB pathway was inhibited after knockout of these three transcription factors (Fig. 4H–J).

### Correlation of *ALOX5* with multiple immune cell types

**Fig. 5:**
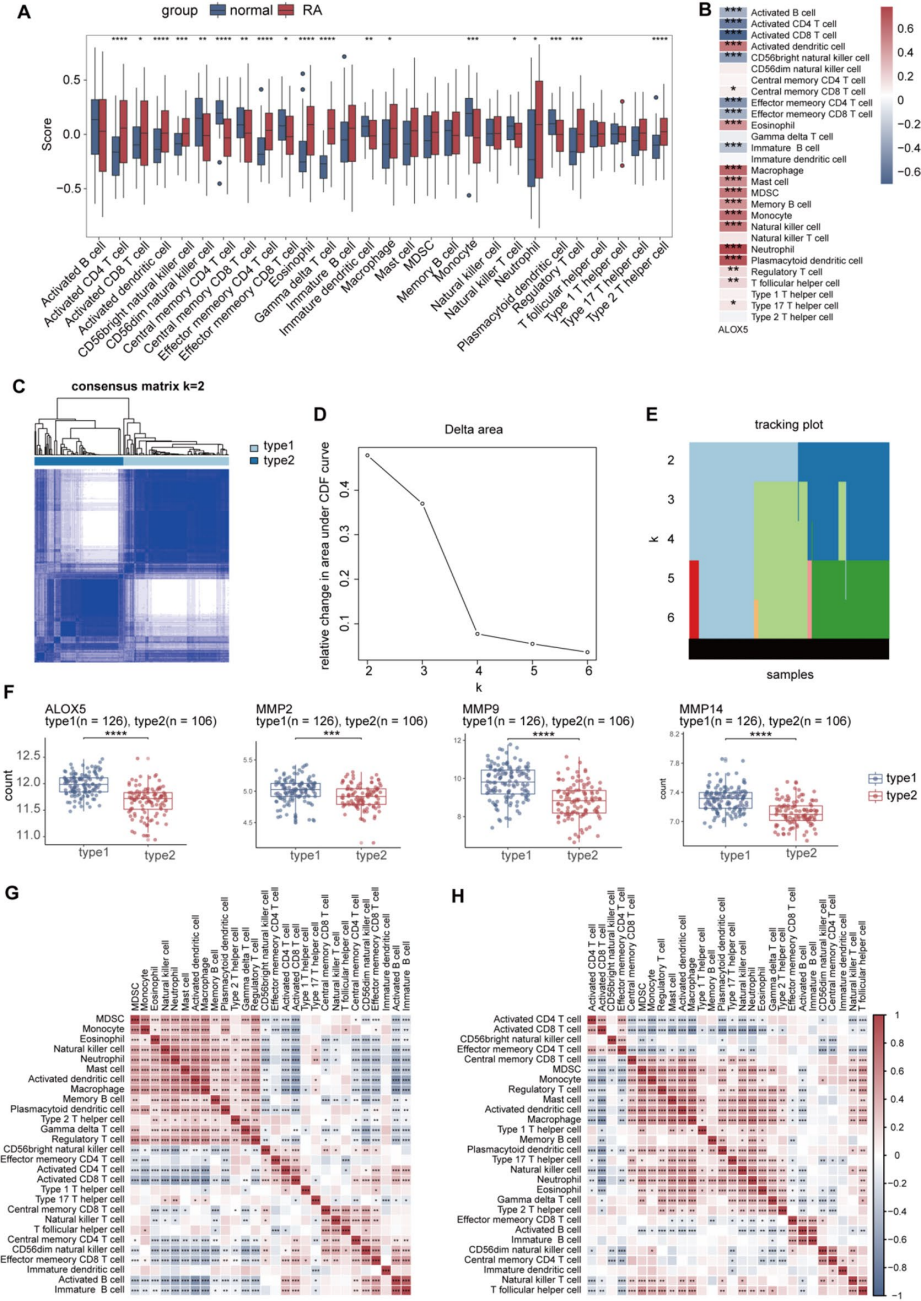
Immune infiltration analysis (*p < 0.05, **p < 0.01,***p < 0.001). (**A**) Differential analysis of immune cells between RA and normal. (**B**) Correlation of *ALOX5* with Immune Cell Types. (**C**) Consensus clustering matrix for k = 2. (**D**) CDF delta area curves. (**E**) Tracking plot. (**F**) Differential analysis of *ALOX5*,*MMP2*,*MMP9*,*MMP14* between subtype 1 and subtype 2. (**G**) Immune Cell Correlation Heatmap for subtype1. (**H**) Immune cell correlation heatmap for subtype2. MDSC cell, Myeloid-Derived Suppressor cell.

**Fig. 6:**
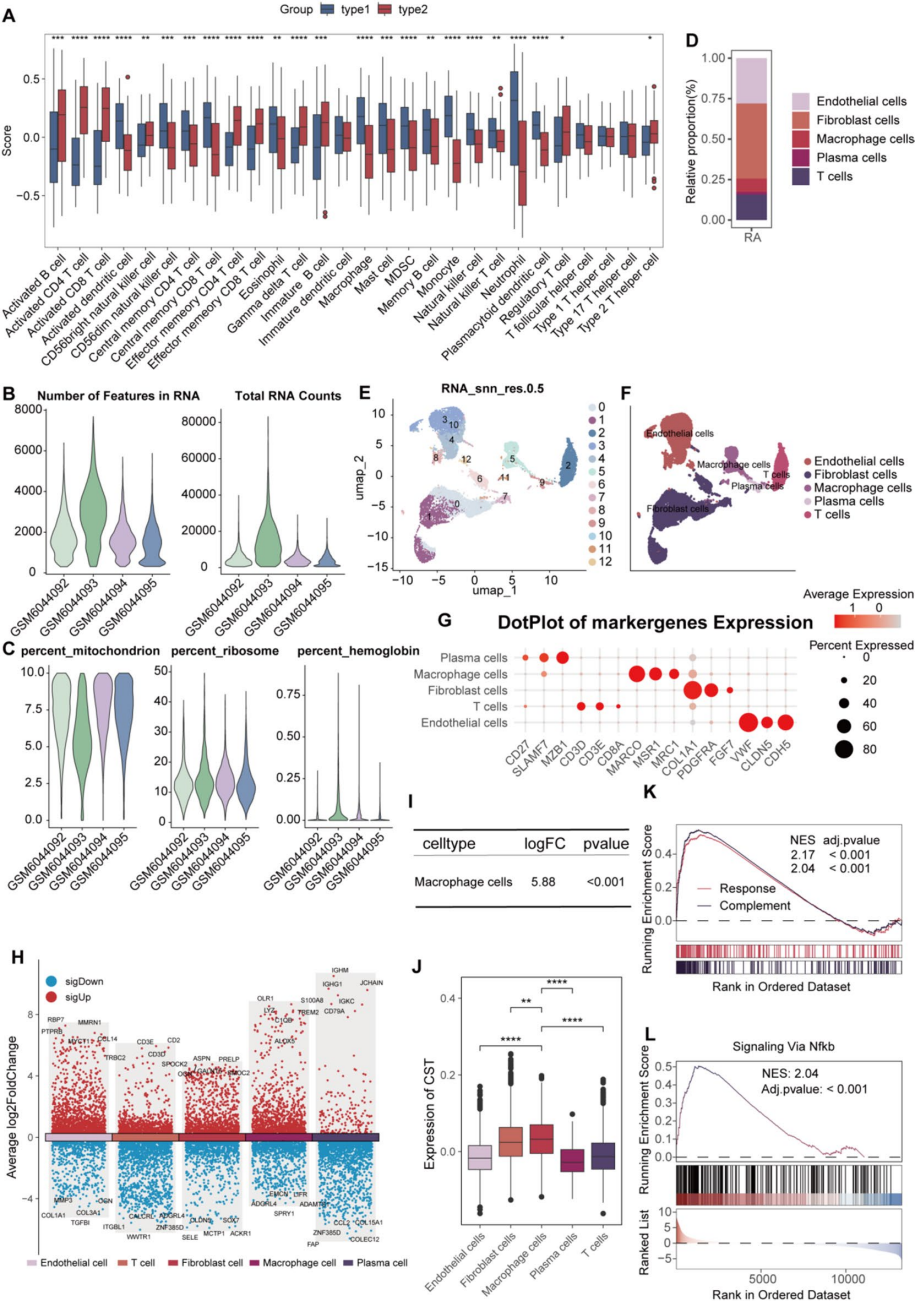
Single cell analysis. (**A**) Differential analysis of immune cells between subtype1 and subtype2. (**B**) The number of features in RA and total RAN counts after quality control. (**C**) The percent of mitochondrion and ribosome, hemoglobin in RA after quality control. (**D**) Cell ratio in RA. (**E**) UMAP visualization of cellular clusters. (**F**) UMAP plot with cell type annotations. (**G**) Bubble Chart for marker genes. (**H**) Multiple volcano maps. (**I**) log2FC for *ALOX5* in differential expression analysis. (**J**) Boxplot of Addomodulescores across different cell types. (**K**) GSEA analysis for differentially expressed genes in macrophages. (**L**) NF-KB pathway. GSEA, Gene Set Enrichment Analysis; GSVA, gene set variation analysis; MDSC cell, Myeloid-Derived Suppressor cell; NFκB, Nuclear Factor kappa-light-chain-enhancer of activated B cells; RA, rheumatoid arthritis; UMAP, Uniform Manifold Approximation and Projection.

Immune infiltration analysis revealed that the scores of immune cells, including activated CD4 T cell, activated dendritic cell and activated CD8 T cell, were significantly higher in the RA group than in the normal group, whereas the scores of CD56dim natural killer cell, central memory CD4 T cell were significantly lower (Fig. 5A). Scatter plots showed that *ALOX5* was significantly positively correlated with various immune cells, such as macrophages, mast cells, and neutrophils, and significantly negatively correlated with activated B cell and activated CD4 T cell(Fig. 5B). Based on the consensus clustering results, the relative changes in the cumulative distribution function (CDF) curves, and the consensus clustering scores, k = 2 was selected as the optimal value to divide the RA samples into two distinct subtypes (Fig. 5C–E). In subtype 2, the expression of *ALOX5*, *MMP2*, *MMP9*, and *MMP14* was significantly reduced (Fig. 5F). Additionally, in type 1, MDSC cells, monocytes, neutrophils, mast cells, and macrophages exhibited stronger correlations among themselves, whereas in type 2, the correlation patterns were altered, with some cells showing different correlation directions and strengths compared to subtype 1, such as MDSC cells with monocytes and regulatory T cell(Fig. 5G,H).Compared to type 1, type 2 exhibited significantly higher expression of activated B cells, activated CD4 T cells, and activated CD8 T cells, whereas macrophages, mast cells, and natural killer cells showed significantly lower expression (Fig. 6A).

### High expression of ALOX5 in macrophages

UMAP clustering analysis of single cells from four RA samples resulted in 12 clusters (Fig. 6B,C), which were annotated into five cell types: plasma cells, macrophages, fibroblasts, T cells, and endothelial cells (Fig. 6D–G). Figure 6H displays the differentially expressed genes (DEGs) among these five cell types, with *ALOX5* significantly overexpressed exclusively in macrophages (Fig. 6I). AddModuleScore quantifies per-cell average expression of a gene set (Seurat 4.0), providing a metric for potential celastrol responsiveness. the AddModuleScore analysis based on triptolide-target genes revealed that macrophages had significantly higher activity scores than the other four cell types (Fig. 6J). GSEA enrichment analysis of DEGs between macrophages and other cells showed significant activation of the complement pathway, inflammatory response pathway (Fig. 6K), and TNF-NFκB pathway (Fig. 6L).

## Discussion

Bone destruction is one of the key pathological features of RA, significantly affecting patients’ quality of life and prognosis^26,27^. In the inflammatory microenvironment, pro-inflammatory cytokines such as TNF-α, IL-6, and IL-1 promote the release of inflammatory factors by macrophages, thereby inducing the expression of RANKL in synovial fibroblasts^28,29^. RANKL binds to its receptor RANK on the surface of osteoclast precursors, activates the NF-κB pathway, and subsequently promotes the differentiation and maturation of osteoclasts^30,31^. Moreover, matrix metalloproteinases (MMPs), particularly MMP-3 and MMP-9, accelerate joint structural destruction in RA joints by degrading collagen and proteoglycans, which are components of articular cartilage and bone matrix^12^. Therefore, inhibiting inflammatory responses and the expression of matrix metalloproteinases is a crucial strategy for delaying bone destruction in rheumatoid arthritis.

While *ALOX5* is known to mediate inflammatory responses, its direct targeting by celastrol has not been established. Here, we identify *ALOX5* as a previously unrecognized direct target of celastrol and further demonstrate that the *ALOX5*–macrophage axis drives bone destruction in rheumatoid arthritis through a novel transcriptional mechanism involving NF-κB/RELA. Celastrol exerts potent anti-inflammatory and antioxidant effects, thereby inhibiting osteoclastogenesis and function, which leads to a reduction in bone destruction, as demonstrated in models of osteoarthritis and osteosarcoma^32,33^. Its mechanism of action involves the modulation of key signaling pathways, including ROS/JNK, NFκB, all of which play critical roles in bone metabolism^34^. These findings highlight celastrol as a promising therapeutic agent for bone-destructive diseases^35,36^. As a member of the lipoxygenase (LOX) family, *ALOX5* is involved in the synthesis of inflammatory mediators and has been implicated in the inflammatory response in RA. Its overexpression may be associated with bone destruction in RA^37^. In this study, 13 key target genes associated with celastrol treatment for RA were systematically identified through differential expression analysis, WGCNA, and network pharmacology. Correlation and multivariate linear regression analyses revealed that high *ALOX5* expression significantly promoted MMP3 protein expression, which is closely linked to the progression of bone destruction in RA^12^, further highlighting the role of *ALOX5* in this process. A predictive model for RA diagnosis and therapeutic evaluation was constructed based on *ALOX5*, other MMPs, and clinical phenotypes. The area under the receiver operating characteristic curve (AUC) was low when only CRP, ESR, TJC28, and SJC28 were used. However, the AUC value increased significantly upon inclusion of *ALOX5*. Further incorporation of *MMP2*, *MMP3*, *MMP9*, and *MMP14*, which showed significant correlation with *ALOX5* expression, elevated the AUC to 0.804.

Molecular docking analysis demonstrated that celastrol can form a stable binding pocket with *ALOX5*. *ALOX5* contains two critical functional domains: the PLAT_LOX domain, which serves as the PLAT domain of 12/15-lipoxygenase and is involved in the direct dioxygenation of phospholipids and cholesterol esters within biological membranes, thereby regulating substrate specificity and the rate-limiting step of catalysis; and the Lipoxygenase domain, which constitutes the catalytic active site of *ALOX5*. This domain mediates the conversion of arachidonic acid into 5-hydroperoxyeicosatetraenoic acid (5-HPEIE), leading to the subsequent formation of leukotriene A4 (LTA4) and ultimately leukotriene B4 (LTB4), both of which are potent pro-inflammatory mediators. These leukotrienes play pivotal roles in inflammatory processes. Celastrol interacts with PRO575, GLU334, and ASP333 residues of *ALOX5* to form the binding pocket, potentially contributing to the inhibition of *ALOX5* enzymatic activity.

Further analysis revealed that *ALOX5* is significantly associated with inflammatory response, complement activation, and the TNF-NFκB signaling pathway in RA. The NF-κB pathway plays a pivotal role in RA pathogenesis, and its aberrant activation has been closely linked to disease progression^38–41^. The NF-κB signaling cascade consists of both canonical and non-canonical pathways. Activation of the canonical pathway begins with signal-induced phosphorylation of IκB proteins by IKK complexes^42^. The IKK complex comprises two homologous catalytic subunits, IKKα and IKKβ, and a regulatory subunit, IKKγ. Activated IKKβ phosphorylates IκB, leading to K48-linked ubiquitination and subsequent proteasomal degradation of IκB. This degradation results in the release of the NF-κB dimer from the cytoplasm, followed by its nuclear translocation and initiation of target gene transcription. Transcription factor analysis identified RElA (p65) as a common transcription factor for both *ALOX5* and *MMP2*, thereby confirming that RElA regulates the expression of *ALOX5* and *MMP2* via the NF-κB signaling pathway^43^.

Gene knockdown analysis using GPSAdb revealed that silencing the common transcription factor of *ALOX5* and *MMP2* led to reduced expression of both *ALOX5* and *MMP2*, accompanied by suppression of the TNF-NFκB signaling pathway. These findings indicate that *ALOX5* plays a significant role in modulating the TNF signaling cascade. Immunoinfiltration analysis demonstrated that *ALOX5* expression was significantly correlated with multiple immune cell types, particularly macrophages, mast cells, MDSCs, and neutrophils, whereas it showed a significant negative correlation with activated B cells, CD4 + T cells and CD8 + T cells. Consensus clustering analysis identified a low-*ALOX5* subgroup (type 2), in which *MMP2*, *MMP9*, and *MMP14* expression levels were significantly downregulated. In contrast, the abundance of activated B cells, CD4 + T cells, and CD8 + T cells was markedly increased, while macrophages, mast cells, and neutrophils were significantly decreased. These results suggest that reduced *ALOX5* expression is associated with altered immune cell infiltration patterns.

Single-cell analysis revealed that *ALOX5* was significantly upregulated in macrophages, and differentially expressed genes associated with *ALOX5* were enriched in pathways related to inflammatory response activation, complement signaling, and TNF-NFκB signaling. AddModuleScore analysis demonstrated that macrophages exhibited significantly higher scores compared to other cell types, suggesting that celastrol primarily exerts its therapeutic effects through interaction with macrophages.

Inflammation plays a critical role in bone destruction in RA, particularly through the action of pro-inflammatory cytokines^44,45^. In this study, *ALOX5* was found to suppress the release of inflammatory cytokines and MMPs, as well as inhibit NF-κB pathway activation.However, all conclusions are based on in silico analyses without experimental validation. Future studies should employ macrophage-specific *ALOX5* knockout or celastrol treatment in collagen-induced arthritis models to directly validate the proposed *ALOX5*–macrophage–NF-κB axis and its role in bone destruction.

Collectively, these findings indicate that celastrol demonstrates significant therapeutic efficacy in RA and holds potential for mitigating RA-associated bone destruction.

## Conclusions

**Fig. 7:**
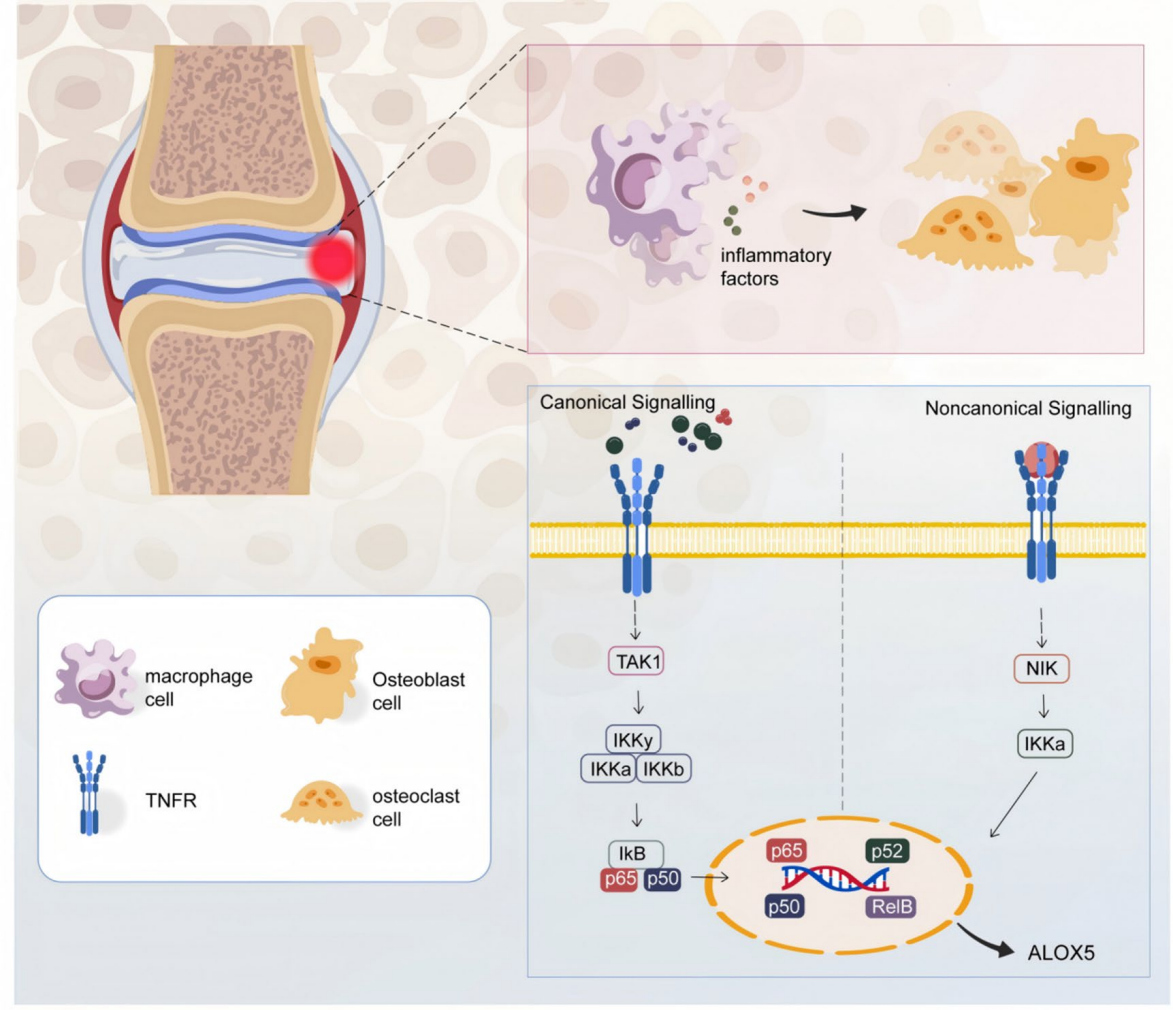
Pathogenic mechanisms of bone destruction in rheumatoid arthritis ^46^ .

In rheumatoid arthritis (RA), the NF-κB signaling pathway is frequently observed to be significantly activated and the reduced NF-κB activity results in decreased production of matrix metalloproteinases, which contributes to the attenuation of joint damage. Celastrol appears to downregulate the expression of the *ALOX5* gene in macrophages, this pattern potentially leads to the suppression of NF-κB pathway activation (Fig. 7). These findings suggest a potential therapeutic role for celastrol in RA management.

## Supplementary Information

Below is the link to the electronic supplementary material.

Supplementary Material 1

## Data Availability

Data is provided within the manuscript or supplementary information files.

## Abbreviations

AUC: Area under the curve
bDMARDs: Biologic disease-modifying antirheumatic drugs
BP: Biological process
CC: Cellular components
CRP: C-reactive protein
CRPDAS28: C-reactive Protein—Disease Activity Score in 28 joints
csDMARDs: Conventional synthetic disease-modifying antirheumatic drugs
CST: Celastrol
DAS28: Disease activity score derivative for 28 joints
DEGs: Differentially expressed genes
ESR: Erythrocyte sedimentation rate
ESRDAS28: Erythrocyte Sedimentation Rate—Disease Activity Score in 28 joints
FDR: False discovery rate
GEO: Gene expression omnibus
GO: Gene ontology enrichment analysis
GS: Gene significance
GSEA: Gene set enrichment analysis
GSVA: Gene set variation analysis
HAQ: Health Assessment Questionnaire
KEGG: Kyoto encyclopedia of genes and genomes
IL-6: Interleukin-6
IL-17: Interleukin-17
MDSC cell: Myeloid-derived suppressor cell
MF: Molecular functions
MM: Module membership
MMPs: Matrix metalloproteinases
MT1-MMP: Membrane-type 1 matrix metalloproteinase
NFκB: Nuclear factor kappa-light-chain-enhancer of activated B cells
PBMCs: Peripheral blood mononuclear cells
PCA: Principal component analysis
RA: Rheumatoid arthritis
RANKL: Receptor activator of nuclear factor-κB ligand
ROC: Receiver operating characteristic
SJC28: Swollen joint count for 28 joints
TJC28: Tender joint counts for 28 joints
TNF-α: Tumor Necrosis Factor-α
TOM: Topological overlap matrix
tsDMARDs: Targeted synthetic disease-modifying antirheumatic drugs
UMAP: Uniform manifold approximation and projection
VAS: Visual analog scale
WGCNA: Weighted gene co-expression network analysis
