# Supplementary material for: Celastrol suppresses bone destruction in rheumatoid arthritis by inhibiting ALOX5 expression in macrophages via the NF-κB pathway
Source: Sci Rep. 2025 Dec 17;16:3137. doi: 10.1038/s41598-025-33001-x (PMC12830925; doi:10.1038/s41598-025-33001-x)
Supplement: Supplementary file 1 — Supplementary Material 1 [file 41598_2025_33001_MOESM1_ESM.docx]

**Supplementary table 1. Details of the datasets.**

| Dataset | GPL Platform | Sample/Details | Year | Data link |
| --- | --- | --- | --- | --- |
| GSE55235 | GPL96 | 10 RA samples and 10 control samples and 10 OA samples | 2014 | https://www.ncbi.nlm.nih.gov/geo/query/acc.cgi?acc=GSE55235 |
| GSE93777 | GPL570 | 232 Whole blood gene expression of rheumatoid arthritis samples and 216 Immune cells gene expression from rheumatoid arthritis and healthy donors samples | 2018 | https://www.ncbi.nlm.nih.gov/geo/query/acc.cgi?acc=GSE93777 |
| GSE200815 | GPL24676 | 4 RA samples and 5 PsA samples | 2022 | https://www.ncbi.nlm.nih.gov/geo/query/acc.cgi?acc=GSE200815 |
| GSE127134 | GPL20301 | RNA-seq on K562 cells treated by CRISPR interference targeting SP1 | 2019 | https://www.ncbi.nlm.nih.gov/geo/query/acc.cgi?acc=GSE127134 |
| GSE127074 | GPL20301 | RNA-seq on K562 cells treated by CRISPR interference targeting RELA | 2019 | https://www.ncbi.nlm.nih.gov/geo/query/acc.cgi?acc=GSE127074 |
| GSE109728 | GPL20795 | RNA-seq of wild type, BCL-XL overexpression, NOXA-/- knockout and p53 knock-down H9 cells and iPS cells treated with mitosis poison nocodazol or MCL1 inhibitor A1210477. RNA-seq of inducible HA-BCLXL overexpression H9 recovered from repeated NOC or AZD treatment. | 2019 | https://www.ncbi.nlm.nih.gov/geo/query/acc.cgi?acc=GSE109786 |
